# Exosomes Derived from Bone Mesenchymal Stem Cells Alleviate Compression-Induced Nucleus Pulposus Cell Apoptosis by Inhibiting Oxidative Stress

**DOI:** 10.1155/2021/2310025

**Published:** 2021-10-25

**Authors:** Yiqiang Hu, Ranyang Tao, Linfang Wang, Lang Chen, Ze Lin, Adriana C. Panayi, Hang Xue, Hui Li, Liming Xiong, Guohui Liu

**Affiliations:** ^1^Department of Orthopedics, Union Hospital, Tongji Medical College, Huazhong University of Science and Technology, Wuhan 430022, China; ^2^Department of Gastrointestinal Surgery, Union Hospital, Tongji Medical College, Huazhong University of Science and Technology, Wuhan 430022, China; ^3^Department of Plastic Surgery, Brigham and Women's Hospital, Harvard Medical School, Boston, MA 02215, USA

## Abstract

Oxidative stress is relevant in compression-induced nucleus pulposus (NP) cell apoptosis and intervertebral disc (IVD) degeneration. Exosomes derived from bone mesenchymal stem cells (BMSCs-Exos) are key secretory products of MSCs, with important roles in tissue regeneration. This research is aimed at studying the protective impact of BMSCs-Exos on NP cell apoptosis caused by compression and investigating the underlying mechanisms. Our results indicated that we isolated BMSCs successfully. Exosomes were isolated from the BMSCs and found to alleviate the inhibitory effect that compression has on proliferation and viability in NP cells, decreasing the toxic effects of compression-induced NP cells. AnnexinV/PI double staining and TUNEL assays indicated that the BMSCs-Exos reduced compression-induced apoptosis. In addition, our research found that BMSCs-Exos suppressed compression-mediated NP oxidative stress by detecting the ROS and malondialdehyde level. Furthermore, BMSCs-Exos increased the mitochondrial membrane potential and alleviated compression-induced mitochondrial damage. These results indicate that BMSCs-Exos alleviate compression-mediated NP apoptosis by suppressing oxidative stress, which may provide a promising cell-free therapy for treating IVD degeneration.

## 1. Introduction

Low back pain (LBP) has emerged as a health issue affecting quality of life and exerting a financial burden on patients and healthcare systems. IVD degeneration is the principal cause of LBP [1, 2]. Therapy of IVD degeneration typically entails conservative treatment or spinal operation to reduce the patient's pain [3, 4]. These treatments are, however, symptomatic and do not cure IVD degeneration. Many complicated factors, including aging and mechanical stress are associated with IVD degeneration. Excessive mechanical loading is regarded as a key cause of IVD degeneration [5, 6]. However, the mechanisms of IVD degeneration have not been completely elucidated.

Oxidative stress is widely considered to be a crucial mechanism of IVD degeneration. Recent studies reported that IVD degeneration is strongly associated with oxidative stress [7, 8]. Although ROS production is important for maintaining intracellular redox homeostasis, excessive ROS production can cause oxidative stress damage leading to cell damage and even death [8, 9]. Growing evidence has revealed that excessive oxidative stress leads to IVD cellular apoptosis, resulting in a reduction in the number of IVD cells. These changes contribute to IVD degeneration [10, 11]. Recent studies have reported that hydrogen peroxide can cause mitochondrial dysfunction and even NP cell death by enhancing oxidative stress, which resulted in IVD degeneration [12]. Previous research also reported that compression induces NP cell apoptosis and decreases mitochondrial membrane potential (MMP) by oxidative stress, leading to IVD degeneration [13]. Based on these studies, searching for an effective means of decreasing the NP cell damage caused by oxidative stress may offer a therapeutic target to treat IVD degeneration.

Stem cell therapy is regarded as an important way for treating several diseases [14, 15]. Interestingly, evidence from prior studies indicated that stem cells can serve their tissue repair function through paracrine exosomes. Exosomes, microvesicles with a diameter between 30 and 150 nm, can deliver growth factors, and proteins to target cells and exert their function [16, 17]. Exosomes have many advantages compared to MSCs, including greater stability and low immunogenicity [18–20]. A recent study reported that MSCs-Exos inhibit TNF-*α-*induced NP apoptosis and restore IVD degeneration [21]. Besides, studies showed that MSCs-Exos ameliorate H_2_O_2_-induced NP cell damage and repair intervertebral disc degeneration via inhibition of oxidative stress [22]. However, the impact of MSCs-Exos on NP death caused by compression remains unclear.

In this research, we isolated BMSCs-Exos and further investigated the protective role of BMSCs-Exos on NP apoptosis induced by compression. Besides, we assessed the impact of oxidative stress in BMSCs-Exos against NP cell apoptosis by measuring ROS level and assessing the function of mitochondria.

## 2. Material and Methods

### 2.1. Isolation and Identification of BMSCs

This study was approved by the Clinical Research Ethics Committee of Tongji Medical College, Huazhong University of Science and Technology. According to our previous research, SD rats were isolated BMSCs [16, 23]. Briefly, the bone marrow of the femur and tibia was washed with a syringe. BMSCs were then incubated in a fresh medium containing 10% depleted exosomes of FBS. After 24 hours, fresh culture media was added. BMSCs were incubated with anti-CD73, CD90, CD34, and CD45 solutions to detect the specific marker. Flow cytometry (Becton Dickinson, USA) was used for testing the labeled cells. The multilineage differentiative capacity was assessed with alizarin red, oil red O, or alcian blue (Cyagen, USA). The results were observed with a microscope (Olympus, Japan).

### 2.2. Isolation and Identification of Exosomes

Ultracentrifugation was applied to isolate and extract BMSCs-Exos. Briefly, the supernatant of BMSCs was collected and centrifuged at 2000 g for 30 minutes. The conditioned medium was centrifuged at 12000 g for 45 minutes and then centrifuged at 100,000 g for 90 minutes. Subsequently, sediment obtained was resuspended with PBS and stored at -80°C. For the validation of exosomes, transmission electron microscopy (TEM) was viewed BMSCs-Exos shape. The size distribution of BMSCs-Exos was tested by nanoparticle tracking analysis (NTA). Specific markers of BMSCs-Exos (CD9, CD63) were detected by western blotting.

### 2.3. Exosome Uptake

A BMSCs-Exos uptake assay was conducted using PKH26 red dye (Sigma-Aldrich, USA). Briefly, BMSCs-Exos were marked with PKH26 according to the instruction book. The NP cells were incubated with PKH26-labeled exosomes. Then, cells were fixed with 4% paraformaldehyde. FITC phalloidin (Solarbio, China) was used to observed labeled the cytoskeleton of NP cells. The NP nuclei were stained with Hoechst 33342. The cells were observed with confocal microscopy (Nikon A1, Japan).

### 2.4. Culture and Treatment of NP Cells

NP cells were obtained and cultured from SD rats (200-250 g) as we described previously [12, 13, 24]. Briefly, after anesthesia with intraperitoneal injection of chloride hydrate, the IVD of rats were harvested immediately. After disinfection, the NP tissue was collected from rat lumbar discs. Then, the obtained NP tissue was digested with 0.25% type II collagenase (Biosharp, China) for 15 minutes. The NP tissue was centrifuged and cultured in DMEM/F-12 (Gibco, USA) containing 10% FBS (Gibco, USA). The NP cells of second generation were used for further experiments. To test the impact of BMSCs-Exos on NP cells, the cells were cultured in a pressure apparatus to simulate mechanical loading conditions of IVD as we described previously [13, 24, 25]. Briefly, the cells received different concentrations of BMSCs-Exos (20, 50, or 100 *μ*g/ml) and received 1.0 MPa compression treatment for 36 hours. Then, the NP cells were used for further experiments. A control group (con) was cultured under the same conditions without compression.

### 2.5. CCK-8 Assay

Cell viability was tested by Cell Counting Kit-8 (CCK-8, Dojindo, Japan) according to the instruction book. In brief, NP cells were cultured in 96-well plates and received different treatments for 36 h, including BMSCs-Exos and compression. After treatment, 100 ml CCK-8 solution containing 90 ml DMEM/F-12 was added in 96-well plates. The cells were hatched at 37°C in the dark. Then, the absorbance at 450 nm was measured by a microplate reader (BioTek, USA).

### 2.6. EdU Incorporation Assay

Cell proliferation was tested by a EdU assay (RiboBio, China) as we used previously [26]. In brief, after receiving different treatments for 36 h, including BMSCs-Exos and compression, the NP cells were assessed with an EdU assay according to the instruction book. NP cells were washed with PBS. Finally, the results of EdU assay were observed with a microscope (Olympus, Japan).

### 2.7. Live/Dead Assay

The live/dead effect of BMSCs-Exos on NP cells induced by compression was assessed by the Calcein-AM/PI probe (Dojindo, Japan). Briefly, after receiving different treatments including BMSCs-Exos and compression for 36 h, the cells were treated with Calcein-AM and PI staining for 20 minutes according to the instruction book. After being washed, the live/dead cells were observed with a microscope (Olympus, Japan).

### 2.8. Lactate Dehydrogenase Release Assay

Lactate dehydrogenase (LDH) release tested the impact of BMSCs-Exos on NP cells induced by compression. After NP cells received different treatments for 36 h, including BMSCs-Exos and compression, the release of LDH from the cells was detected by the LDH assay according to the instruction book. Absorbance at 490 nm was measured by a microplate reader (BioTek, USA).

### 2.9. Annexin V/PI Staining

The Annexin V-FITC/PI apoptosis assay (KeyGen Biotech, China) was detected the apoptotic impact of BMSCs-Exos on NP cells induced by compression according to the instruction book. In brief, after receiving different treatments including BMSCs-Exos and compression for 36 h, the NP cells were digested by trypsinization and collected. The cells were resuspended in a binding buffer. Subsequently, Annexin V and PI staining were added in NP cells for 15 minutes. Cells were tested by flow cytometry (Becton Dickinson, USA).

### 2.10. TUNEL Staining

TUNEL assays tested the apoptosis of NP cells according to the instruction book. Briefly, after being received different treatments for 36 h, including BMSCs-Exos and compression, the cells were fixed with 4% paraformaldehyde. Cells were washed and were permeabilized with TritonX-100 for 10 minutes. Then, cells were treated with TUNEL staining (Roche, Germany). The cells were stained with DAPI. The results were observed with a microscope (Olympus, Japan).

### 2.11. Measurement of Cellular ROS

DCFH-DA assay (Nanjing Jiancheng, China) tested the intracellular ROS level according to the instruction book. In brief, after being treated with compression and BMSCs-Exos for 36 h, NP cells were digested by trypsinization and collected. Subsequently, cells were incubated with DCFH-DA at 37°C. Cells were tested by flow cytometry (Becton Dickinson, USA).

### 2.12. MDA Assay

The MDA concentration of cells was tested by a Lipid Peroxidation MDA Assay (Beyotime). Briefly, after NP cells received different treatments for 36 h, including compression and BMSCs-Exos, the cells were lysed and then centrifuged at. MDA solution was added in the supernatant according to the instruction book. Absorbance at 532 nm was tested by a microplate reader (BioTek, USA).

### 2.13. JC-1 Staining

JC-1 staining assay (Beyotime, China) tested NP cell mitochondrial membrane potential (MMP) according to the instruction book. Briefly, after being treated with compression and BMSCs-Exos for 36 h, NP cells were digested and collected. Subsequently, cells were stained with a JC-1 fluorescent probe at 37°C for 20 minutes. Cells were centrifuged and resuspended in a staining buffer. Cells were tested by flow cytometry (Becton Dickinson, USA). Besides, MMP was also tested by a fluorescence microscope (Olympus, Japan).

### 2.14. Mitotracker Staining

The mitochondria of NP cells were detected with mitotracker staining (ThermoFisher, USA) according to the instruction book. Briefly, after receiving different treatments including compression and BMSCs-Exos for 36 h, NP cells were washed twice and incubated with a mitotracker staining probe. Subsequently, the cells were counterstained with Hoechst 33342. After being washed, cells were observed in the dark by a microscope (Olympus, Japan).

### 2.15. Statistical Analysis

GraphPad Prism (GraphPad Software Inc, USA) was used for statistical analysis. Data were expressed as mean ± standard deviation (SD) from three independent experiments. Differences between groups were analyzed by Student's *t*-test and the ANOVA test. Differences in more than two groups were analyzed by Tukey's multiple comparison test. *P* < 0.05 was considered statistically significant.

## 3. Results

### 3.1. Identification of BMSCs

**Figure 1:**
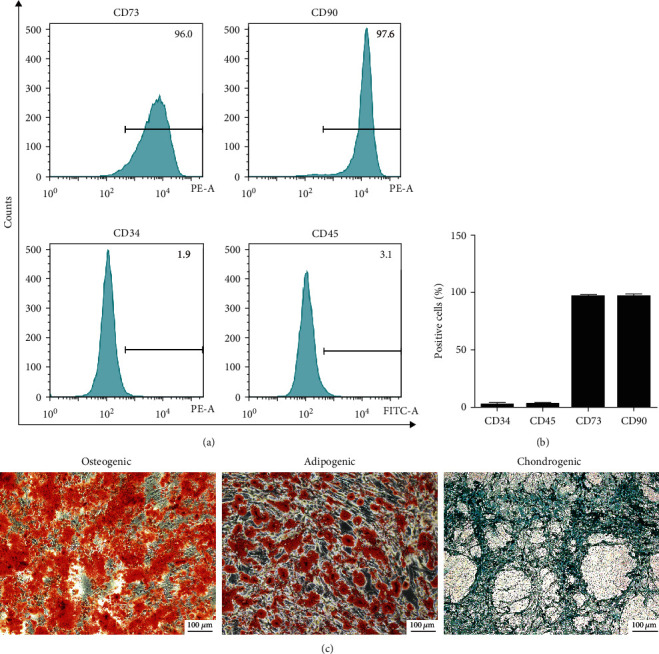
Identification of BMSCs. (a) Flow cytometry tested the CD34, CD45, CD73, and CD90 surface markers on the BMSCs. (b) Quantification analysis of positive cells in BMSCs. (c) Alizarin red, oil red O, and alcian blue staining tested osteogenic, adipogenic, and chondrogenic differentiation of BMSCs, respectively. Scale bar: 100 *μ*m.

BMSCs were isolated and cultured from rat bone marrow. Flow cytometry was used for surface marker detection on the BMSCs, demonstrating that the isolated cells highly expressed specificity surface markers of stem cells, including CD73 and CD90. The positive rates of the cells were greater than 95%. Our results indicated that these cells lowly expressed CD34 and CD45 (Figures 1(a) and 1(b)). The multilineage differentiation of these cells was detected using a BMSCs-osteogenic, adipogenic, and chondrogenic differentiation medium. Our results revealed that the cells with osteogenic induction formed multiple calcium deposits, visualized with alizarin red staining. Oil droplets were formed in the BMSCs with oil red O staining after treating the cells with an adipogenic differentiation medium. After treatment with a chondrogenic differentiation medium, the cells produced acidic mucopolysaccharide visualized with alcian blue (Figure 1(c)). Together, our results displayed that the cells isolated met the criteria of MSCs as described by International Society for CellularTherapy.

### 3.2. Characterization of BMSCs-Exos

**Figure 2:**
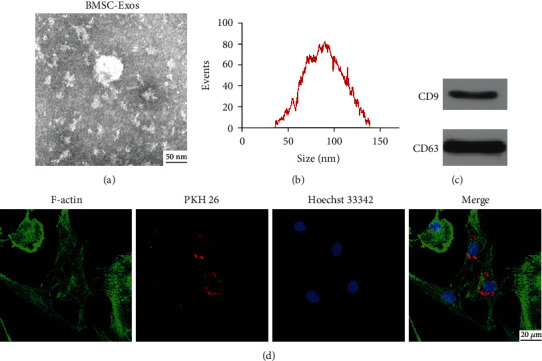
Characterization of BMSC-Exos. (a) The exosome morphology was observed by TEM. Scale bar: 50 nm. (b) Size distribution of BMSC-Exos was examined with NTA. (c) The exosomal marker proteins CD9 and CD63 of BMSC-Exos were tested by western blotting. (d) The assay of BMSC-Exos uptake by NP cells was observed by laser scanning confocal microscopy. The exosomes, cytoskeleton, and cell nucleus are displayed red, green, and blue, respectively. Scale bar: 20 *μ*m.

Ultracentrifugation was used to isolate and extract BMSCs-Exos from the medium of BMSCs to test the impact of BMSCs-Exos on NP cells. The exosomes isolated displayed spherical shape and membrane vesicles from the result of TEM (Figure 2(a)). The size distribution of exosomes was detected by NTA, identifying that the exosomes mainly ranged from 30 to 150 nm (Figure 2(b)). Western blotting was tested specificity markers, showing that the exosomes derived from BMSCs expressed specificity markers of exosomes, such as CD9 and CD63 (Figure 2(c)). These data showed that the isolated exosomes possessed exosomal characteristics. In addition, PKH26 probing assessed the exosomal uptake by NP cells. After labeling the exosomes with PKH26, confocal microscopy revealed that the BMSCs-Exos were endocytosed by NP cells (Figure 2(d)).

### 3.3. BMSCs-Exos Alleviate Compression-Induced Cytotoxicity in NP Cells

**Figure 3:**
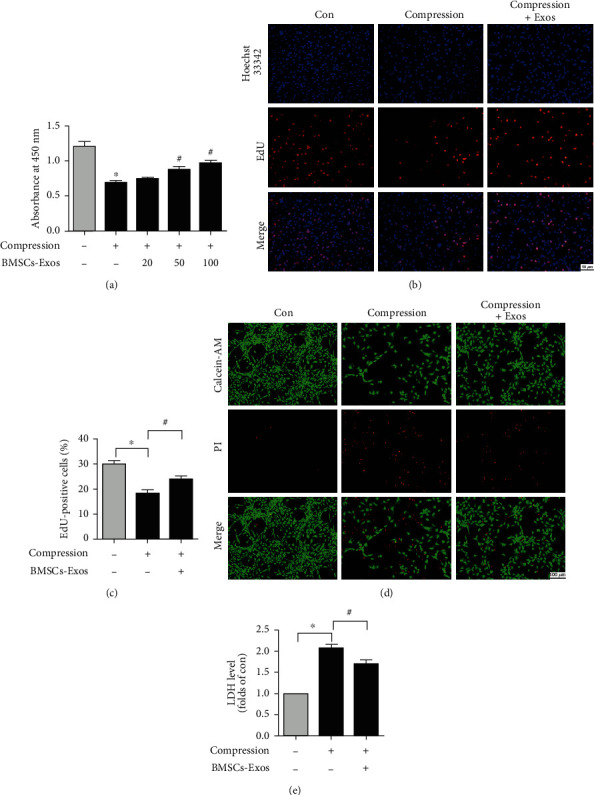
BMSC-Exos alleviate compression-caused cytotoxicity in NP cells. (a) CCK-8 assay tested the impact of BMSC-Exos (0, 20, 50, and 100 *μ*g/ml) and compression on NP cell viability. (b) The NP cell proliferation was tested by an EdU incorporation assay. The EdU-positive cells displayed red fluorescence. Scale bar: 50 nm. (c) Quantification analysis percentage of EdU-positive cells. (d) Calcein-AM/PI staining tested the cytotoxicity effect of BMSC-Exos on NP cells caused by compression. Scale bar: 100 nm. (e) The released result of LDH in NP cells. Data are expressed as the mean ± SD from three independent experiments. ^∗^*P* < 0.05 versus the control group; ^#^*P* < 0.05 versus the compression group.

CCK-8 assay tested the impact of BMSCs-Exos on NP cell viability. The cell viability was significantly decreased by compression. BMSCs-Exos significantly attenuated the suppressive impact of compression on NP cell viability. The protective impact of BMSCs-Exos was greatest at a dose of 100 *μ*g/ml; therefore, 100 *μ*g/ml BMSCs-Exos was employed in subsequent experiments (Figure 3(a)). The impact of BMSCs-Exos on NP cell proliferation was tested by an EdU incorporation assay. The results demonstrated that the EdU-positive cells in BMSCs-Exos groups was greater than that in the compression groups alone (Figures 3(b) and 3(c)). To test the protective impact of BMSCs-Exos on NP cells, Calcein-AM/PI probing tested the cytotoxicity effect of BMSCs-Exos or compression on NP cells. The results showed that live cells (green fluorescence) were greater in the BMSCs-Exos groups while dead cells (red fluorescence) were higher in the compression group (Figure 3(d)). Subsequently, LDH assay tested the toxicity effect of NP cells. We found that the release of LDH was increased in the compression group, while the release of LDH was alleviated by BMSCs-Exos compared with the compression group (Figure 3(e)). Overall, BMSCs-Exos relieved compression-induced NP cell cytotoxicity.

### 3.4. BMSCs-Exos Reduced Compression-Caused NP Cell Apoptosis

**Figure 4:**
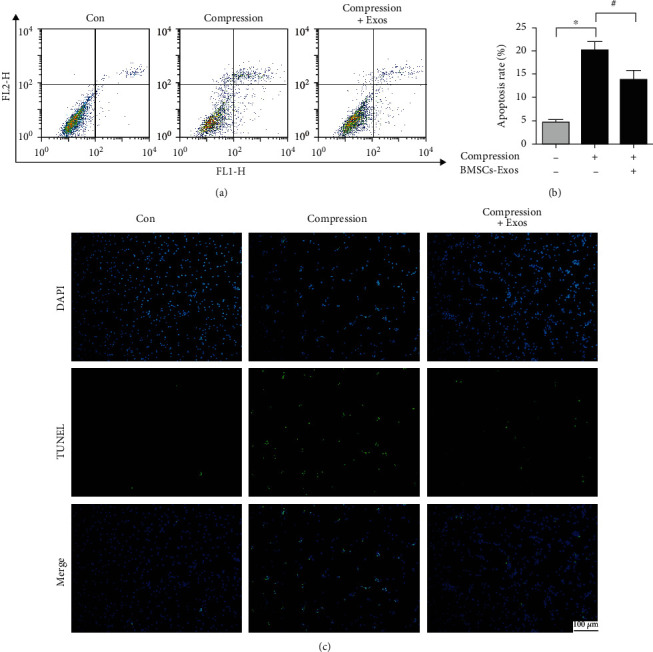
BMSC-Exos reduced compression-caused apoptosis in NP cells. (a) Annexin V/PI staining tested NP cell apoptosis by flow cytometry. (b) Quantification analysis of NP cell apoptosis. (c) The TUNEL staining observed NP cell apoptotic changes. Scale bar: 100 nm. Data are expressed as the mean ± SD from three independent experiments. ^∗^*P* < 0.05 versus the control group; ^#^*P* < 0.05 versus the compression group.

To test the impact of BMSCs-Exos on cell apoptosis caused by compression, Annexin V/PI staining detected NP cell apoptosis. Flow cytometry revealed that compression significantly increased NP cell apoptosis. BMSCs-Exos significantly alleviated NP cell apoptosis caused by compression (Figures 4(a) and 4(b)). In addition, we also evaluated the NP cell apoptosis by TUNEL assays. We found that the number of TUNEL-positive cells (green fluorescence) in compression groups was increased compared with the control groups, while BMSCs-Exos decreased the number of TUNEL-positive cells induced by compression (Figure 4(c)). Our research indicated that BMSCs-Exos reduced compression-induced cell apoptosis in NP cells.

### 3.5. BMSCs-Exos Decreased Compression-Caused Oxidative Stress in NP Cells

**Figure 5:**
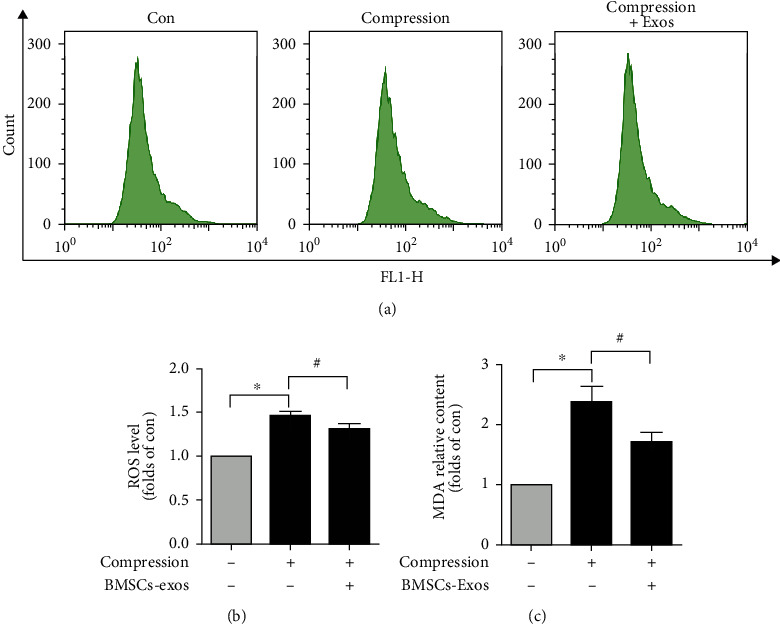
BMSC-Exos decreased compression-induced oxidative stress in NP cells. (a) DCFH-DA probes tested NP cell ROS level by flow cytometry. (b) Quantification analysis of ROS level in NP cells. (c) MDA assay was tested MDA levels. Data are presented as the mean ± SD from three independent experiments. ^∗^*P* < 0.05 versus the control group; ^#^*P* < 0.05 versus the compression group.

Growing evidences have revealed that mitochondrial dysfunction leads to cell oxidative stress and apoptosis. To explore the impact of BMSCs-Exos on NP cell oxidative stress caused by compression, DCFH-DA fluorescent probes examined ROS level. Flow cytometry revealed that compression promoted NP cell ROS production and caused oxidative stress, while ROS level of NP cells in BMSCs-Exos groups was markedly decreased compared with compression groups alone (Figures 5(a) and 5(b)). To further test the suppressive impact of BMSCs-Exos on oxidative stress, we used MDA assay to further investigate the oxidative stress level. We found that compression significantly increased the MDA content in NP cells, while BMSCs-Exos observably decreased the MDA level induced by compression (Figure 5(c)). Our research showed that BMSCs-Exos decreased compression-induced oxidative stress in NP cells.

### 3.6. BMSCs-Exos Protected against Compression-Caused Mitochondrial Damage in NP Cells

**Figure 6:**
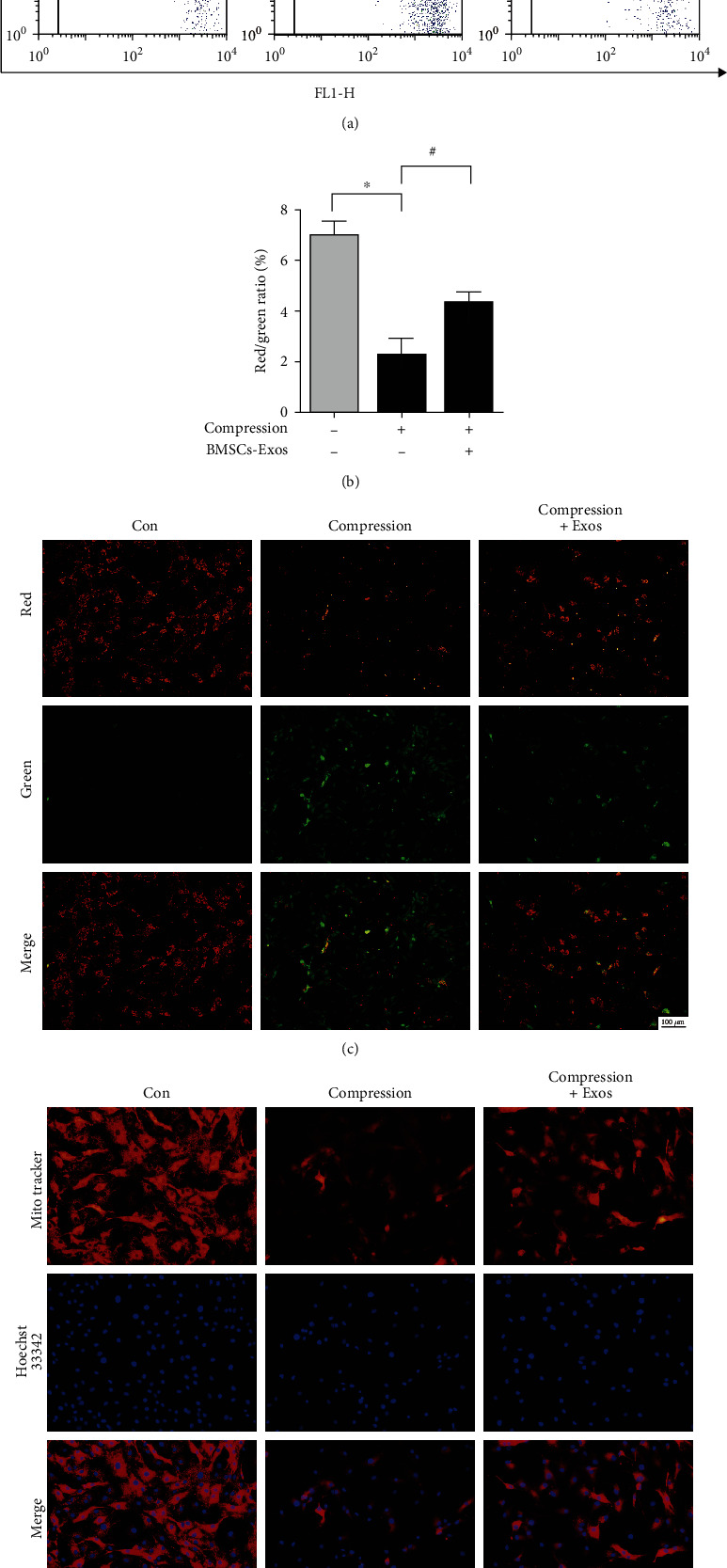
BMSC-Exos protected against compression-induced mitochondrial damage in NP cells. (a) JC-1 staining tested MMP by flow cytometry. (b) Quantification analysis of JC-1 assay. (c) JC-1 staining was tested under a fluorescence microscope in NP cells. Scale bar: 100 nm. (d) Mitotracker staining tested cell mitochondria. Scale bar: 50 nm. Data are presented as the mean ± SD from three independent experiments. ^∗^*P* < 0.05 versus the control group; ^#^*P* < 0.05 versus the compression group.

To study the protective impact of BMSCs-Exos on mitochondrial damage caused by compression in NP cells, flow cytometric analysis was tested MMP by JC-1 staining. The results displayed that compression significantly decreased the red/green ratio in NP cells compared with the control group, and the ratio of red to green in the BMSCs-Exos group was significantly increased than that in the control group (Figures 6(a) and 6(b)). In addition, we observe the protective effects of BMSCs-Exos on MMP by a fluorescence microscope; our results demonstrated that normal NP cells of the control group mainly showed red fluorescence. However, the cells in the compression group exhibited more green fluorescence while faint red fluorescence. BMSCs-Exos improve red fluorescence inhibited by compression (Figure 6(c)). Besides, we further used mitotracker staining to show the impact of BMSCs-Exos on NP cell mitochondria. The results of the fluorescence microscope revealed that compression obviously decreased intracellular mitochondria compared with the control group, while BMSCs-Exos protected against compression-induced intracellular mitochondria (Figure 6(d)). Those results demonstrated that BMSCs-Exos protected against compression-induced mitochondrial damage in NP cells.

## 4. Discussion

In this study, we firstly detected whether BMSCs-Exos protected NP cell apoptosis caused by compression and the possible molecular mechanisms. We successfully isolated BMSCs-Exos and found that BMSCs-Exos reduced the suppressive impact of compression on viability and proliferation of NP cells. Our research revealed that BMSCs-Exos reduced NP cell cytotoxicity caused by compression. BMSCs-Exos also relieved compression-mediated NP cell apoptosis by inhibiting oxidative stress and mitochondrial damage.

Low back pain is one of the most prevalent bone diseases around the world. IVD degeneration is an essential reason for LBP. Growing evidence has revealed that mechanical compression is a principal cause of IVD degeneration [27, 28]. IVDs experience various mechanical loads during daily activities. Recent studies have displayed that mechanical loading reduced the activity and number of IVD cells, leading to IVD degeneration [29]. Excessive IVD cell death under a compressed microenvironment makes it difficult to maintain IVD activity and number [30]. The unfavorable factors can result in IVD degeneration. Huang et al. showed that compression induces senescence of NP cells and contributes to IVD degeneration [31]. Based on this research, maintenance of the number and viability of IVD cells is considered an important therapeutic strategy for IVD degeneration.

Exosomes secreted by MSCs are critical secretion of MSCs which can mediate cell communication between MSCs and other cells. Recent studies displayed that exosomes are a treatment modality that may offer a promising therapeutic strategy for tissue regeneration [32, 33]. Exosomes have received extensive attention because they were reported to possess functional proteins and miRNAs that have many positive impacts in mediating cellular function and treating a variety of diseases [16, 34, 35]. Recent studies indicated that MSCs-Exos protect endplate chondrocytes against death and offer a potential strategy for IVD degeneration [36]. Zhang et al. reported that MSC-derived Exos ameliorate LPS-induced cell pyroptosis, treating IVD degeneration [37]. In our study, we found that BMSCs-Exos alleviated the suppressive impact of compression on NP cell viability. BMSCs-Exos also promoted NP cell proliferation inhibited by compression. In addition, our results displayed that BMSCs-Exos alleviated cytotoxicity caused by compression in NP cells.

Cell apoptosis plays a crucial role in the clearance of damaged or nonessential cells. Many studies recently reported that apoptosis is considered a crucial way of IVD cell death and plays an essential role in IVD degeneration process [26, 38]. Our previous research showed that compression increased NP cell apoptosis, causing IVD degeneration [13]. Lin et al. showed that edaravone could ameliorate NP cell cytotoxicity and apoptosis caused by compression, highlighting a potential treatment of IVD degeneration [24]. Recent research has reported that regulating TIGAR improved compression-induced NP cell apoptosis, promoting IVD degeneration repair [25]. Interestingly, Zhu et al. reported that MSC-derived exosomes ameliorate interleukin-1*β*-induced NP cell apoptosis. They support that MSC-derived exosomes have therapeutic potential for IVD degeneration diseases [39]. In our research, our results showed that BMSCs-Exos alleviate compression-induced cell apoptosis in NP cells.

Multiple studies displayed that apoptotic signals trigger the mitochondrial dysfunction resulting in an increase in ROS production and establishment of an oxidative stress environment. Oxidative stress caused cell death in IVD degeneration [26, 28]. Recent researchers reported that excess ROS caused mitochondria and DNA damage, which leads to cell injury and even death [24, 40]. Recent research has reported that CsA relieved NP stem cell apoptosis mediated by compression via alleviating oxidative stress and mitochondrial dysfunction, which suggested that CsA is a potential way for IVD degeneration [40]. Chen et al. reported oxidative stress played a vital role in NP cell apoptosis caused by compression. They support that regulation of cell death such as necroptosis and apoptosis may contribute to NP cell survival and repair IVD degeneration [28]. In addition, exosomes from adipose stem cells alleviated oxidative stress in macrophages to promote tissue repair and regeneration [41]. Exosomes derived from MSCs ameliorate H_2_O_2_-caused mitochondrial dysfunction and prevent the progression of IVD degeneration by inhibiting inflammation [22]. In our study, we found BMSCs-Exos decreased compression-caused NP cell oxidative stress by decreasing ROS production. BMSCs-Exos also protected against compression-decreased MMP and induced mitochondrial damage in NP cells.

There are several limitations in our research. First, all of our experiments were performed in *vitro*, and the results may not necessarily be indicative of what occurs in *vivo*. Second, this study focused on the protective impact of BMSCs-Exos on NP cell apoptosis caused by compression through inhibition of oxidative stress. We will investigate how BMSCs-Exos exert their protective effect in compression-induced oxidative stress in the future.

## 5. Conclusions

In this study, we successfully isolated exosomes from BMSCs and found that BMSCs-Exos alleviate NP cell viability caused by compression. The results indicated that BMSCs-Exos inhibited compression-induced cell cytotoxicity and apoptosis. The underlying protective mechanism of BMSCs-Exos on NP cell apoptosis-induced compression was linked to NP cell oxidative stress. Taken together, these findings may provide a promising cell-free therapy for treating IVD degeneration.

## Data Availability

The data used to support the findings of this study are available from the corresponding author upon request.
